# MicroRNAs in metabolism for precision treatment of lung cancer

**DOI:** 10.1186/s11658-024-00632-3

**Published:** 2024-09-10

**Authors:** Giovanna Carrà, Jessica Petiti, Federico Tolino, Rita Vacca, Francesca Orso

**Affiliations:** 1 Department of Clinical and Biological Sciences, University of Turin, Turin, Italy; 2 San Luigi Gonzaga Hospital, Orbassano, Italy; 3 Division of Advanced Materials Metrology and Life Sciences, Istituto Nazionale di Ricerca Metrologica (INRiM), 10135 Turin, Italy; 4 Department of Translational Medicine (DIMET), University of Eastern Piedmont, Novara, Italy; 5 Molecular Biotechnology Center “Guido Tarone”, University of Torino, Turin, Italy

**Keywords:** Lung cancer, Metabolism, Metabolic reprogramming, miRNAs

## Abstract

The dysregulation of miRNAs in lung cancer has been extensively documented, with specific miRNAs acting as both tumor suppressors and oncogenes, depending on their target genes. Recent research has unveiled the regulatory roles of miRNAs in key metabolic pathways, such as glycolysis, the tricarboxylic acid cycle, fatty acid metabolism, and autophagy, which collectively contribute to the aberrant energy metabolism characteristic of cancer cells. Furthermore, miRNAs are increasingly recognized as critical modulators of the tumor microenvironment, impacting immune response and angiogenesis. This review embarks on a comprehensive journey into the world of miRNAs, unraveling their multifaceted roles, and more notably, their emerging significance in the context of cancer, with a particular focus on lung cancer. As we navigate this extensive terrain, we will explore the fascinating realm of miRNA-mediated metabolic rewiring, a phenomenon that plays a pivotal role in the progression of lung cancer and holds promise in the development of novel therapeutic strategies.

## Introduction

MicroRNAs (miRNAs) have emerged as powerful regulators in the intricate landscape of gene expression, orchestrating a symphony of molecular events that underpin fundamental biological processes. While compact in size and structure, miRNA role in gene regulation carries substantial significance, shaping the destiny of cells and tissues [1].

To understand the significance of miRNAs in gene regulation, we must first appreciate their fundamental nature. miRNAs are small, noncoding RNA molecules typically composed of 20–25 nucleotides [2]. miRNAs are predominantly situated within the introns or exons of protein-coding genes, as well as in intergenic regions. These genomic *loci* are transcribed within the nucleus by RNA polymerase II, resulting in the generation of a lengthy primary miRNA (pri-miRNA). Subsequently, the DROSHA-DGCR8 complex processes the pri-miRNA to release a precursor miRNA (pre-miRNA). Exportin 5 facilitates the translocation of the pre-miRNA to the cytoplasm, where the RNase III enzyme Dicer cleaves it, giving rise to a mature miRNA. Ultimately, the mature miRNA is loaded onto Argonaute family (AGO) proteins, forming the functional RNA-induced silencing complex (RISC) that reduces target gene expression [3]. This nuanced regulatory mechanism allows miRNAs to impact a wide array of biological processes, including cell proliferation, differentiation, apoptosis, and immune responses [4–6].

The emerging role of miRNAs in cancer has shed light on their significance in the intricate network of oncogenesis and tumor progression. Cancer is fundamentally characterized by uncontrolled cell growth and aberrant gene expression. miRNAs, capable of modulating gene expression patterns, are frequently found to be dysregulated in various cancer types. These aberrations can serve as both drivers and suppressors of carcinogenesis, highlighting the dual role of miRNAs in cancer biology [7].

In particular, lung cancer (LC), a significant global health challenge, stands as a striking example of the interplay between miRNAs and cancer [7]. LC encompasses a diverse group of malignancies primarily affecting the respiratory system. Among its primary subtypes, non-small cell lung cancer (NSCLC) and small cell lung cancer (SCLC) are the most prevalent, each characterized by unique clinical features and molecular profiles. Despite advances in diagnosis and therapy, LC remains a leading cause of cancer-related deaths worldwide [8].

Within the panorama of LC, the role of miRNAs is gaining increasing recognition. MiRNAs are strictly involved in LC initiation, progression, metastasis, and drug resistance. They influence critical processes such as epithelial-mesenchymal transition (EMT), angiogenesis, and immune evasion, all contributing to the complex pathophysiology of LC [9–11]. Also in this context, miRNAs represent a double-edged sword, capable of either promoting or suppressing lung tumorigenesis depending on the specific miRNA and its targets.

As we delve deeper into the intricate tangle of miRNA-mediated gene regulation, we encounter a captivating dimension that has evolved into a mainstream paradigm in cancer research: metabolic rewiring. Metabolism, encompassing all biochemical reactions within a cell, is fundamental to cellular function and is tightly regulated. In tumor cells, this regulation is disrupted, resulting in altered metabolic pathways that confer a survival advantage [12].

MiRNAs have emerged as master coordinators in this metabolic orchestra, dictating the fate of metabolic enzymes, transporters, and signaling molecules [13–15]. The consequences of miRNA-mediated metabolic rewiring extend far beyond the scope of energy production and nutrient utilization: they intricately intertwine with cancer cell proliferation, evasion of cell death, and resistance to therapy. Thus, understanding the connections between miRNA dysregulation, metabolic rewiring, and LC progression holds huge promise for both unraveling the mysteries of this devastating disease and designing innovative therapeutic interventions.

In this extensive exploration, we embark on a comprehensive journey that traverses the landscape of miRNAs, gene regulation, and cancer biology. We will explore the mechanisms by which miRNAs exert their influence on gene expression, uncover their emerging roles in the context of LC, and illuminate the transformative potential of miRNA-mediated metabolic rewiring in shaping the future of LC therapy.

## miRNA regulation of glycolytic pathways

Most cancer cells exhibit increased glycolysis and reduced mitochondrial oxidative phosphorylation, enabling them to produce ATP as their primary energy source. This phenomenon is commonly referred to as the Warburg effect [16]. Perhaps due to its prevalence as an energy source or being one of the earliest metabolic mechanisms identified in cancer cells, the role of miRNAs in glucose metabolism appears to be more clearly defined than in other contexts.

The list of miRNAs found to be dysregulated and related to glucose metabolism is numerous. For example, a substantial decrease in the expression of miR‑133b has been identified in radioresistant NSCLC cells. This miRNA plays a pivotal role in inhibiting glycolysis within LC cells by targeting pyruvate kinase isoenzyme M2, PKM2, a critical enzyme involved in glycolysis. This study marks the pioneering demonstration of the significant suppression of glucose metabolism in LC cells by miR‑133b, revealing a novel mechanism through which miR‑133b functions as a tumor suppressor [17].

As mentioned above, but also investigated in earliest studies, chemo- and radiotherapy-resistant cancer cells exhibit a higher rate of anaerobic glycolysis when compared with their parental counterparts [18]. Among glycolytic enzymes, hexokinase (HK) plays a primary role, since it is highly expressed in cancer cells, and is linked to poor overall survival in patients with cancer [19, 20]. In this context, Xin et al. revealed that radiation induces the expression of miR-155, and its overexpression in LC cells is linked to increased radio-resistance. Moreover, suppressing miR-155 through antisense transfection could potentially sensitize LC cells to radiation. These findings suggest a possible association between miR-155 and radio-resistance, thereby supporting the oncogenic properties of miR-155 in NSCLC. Furthermore, the study unveiled a pivotal role for the miR-155-HK2-glycolysis axis [21].

A significant downregulation of miR‐33b was observed in NSCLC cells/tissue. miR‐33b overexpression not only inhibits the growth of NSCLC cells, but also reduces glucose metabolism. This study reveals an inverse relationship between miR‐33b levels and the rate of glucose metabolism, with higher miR‐33b expression leading to a reduced rate of glucose metabolism. Additionally, the research suggests that the suppression of NSCLC cell growth by miR‐33b inhibition may be mediated by its targeting of lactate dehydrogenase (*LDHA*). Moreover, miR‐33b was identified as a regulator of glucose metabolism in NSCLC cells through its interaction with *LDHA*. In summary, miR‐33b emerges as an anti-NSCLC molecule, reshaping glucose metabolism by targeting *LDHA* [22].

Glucose transporter 1 (GLUT1) functions as a glucose transporter, serving as the initial rate-limiting step in cellular glucose metabolism by facilitating glucose influx into the cell [23]. HKII is a pivotal enzyme responsible for catalyzing the first step in the glycolysis pathway. These two proteins play crucial roles in regulating cellular glycolysis and overall energy metabolism [24]. Among the studies investigating the role of miRNAs in relation to glucose metabolism, many of them focus on the regulation of GLUT1. De-bin Ma et al. highlighted that miR-6077 suppresses GLUT1 expression and enhances the sensitivity of patient-derived lung adenocarcinoma (AC) cells to anlotinib [25]. Similarly, research has delved into the effects of miR-199a-5p and its target gene *GLUT1* (*SLC2A1*) on various aspects of NSCLC, including cell proliferation, migration, cell cycle regulation, and apoptosis, identifying a possible new molecular target and perhaps a marker for early diagnosis in the context of NSCLC [26].

It has been unveiled that miR‑144 assumes a crucial role in orchestrating cellular metabolism. When miR‑144 is suppressed or knocked down, it leads to alterations in glucose uptake and secretion within the cells. Consequently, the influence of miR‑144 on glucose uptake and secretion becomes apparent in the metabolic processes. Additionally, this study has established that miR‑144 plays a pivotal role in regulating cell proliferation, further emphasizing its significance. GLUT1, a key component regulated by miR‑144, was found to be upregulated in LC. This enhanced expression of GLUT1 is intricately linked with miR‑144, and it exerts a substantial impact on the cellular metabolic pathways. This study provides an extensive and comprehensive understanding of the intricate relationship between miR‑144 and cellular metabolism [27].

In a unique study, it has been shown that miR-124 exerts a negative regulatory effect on the expression of both GLUT1 and HKII in NSCLC cells, resulting in reduced glucose consumption and lactate production. Specifically, overexpression of miR-124 negatively regulates AKT1 and AKT2 and impacts the expression of GLUT1 and HKII. Ultimately, this leads to the suppression of energy metabolism in tumor cells [28].

Another key enzyme in glucose metabolism, finely regulated by a complex network including not only enzymes and metabolites but also miRNAs, is phosphofructokinase (PFK) [29, 30]. In LC cells, it has been demonstrated that miR-128 exerts regulatory control over PFKL by inhibiting AKT phosphorylation. This study elucidates the existence of a novel miR-128-PFKL-AKT axis within LC cells, which orchestrates a metabolic shift affecting glucose consumption, lactate production, and ATP generation, consequently promoting cell proliferation and anchorage-independent growth. The research also establishes an inverse relationship between miR-128 levels and *PFKL* mRNA expression in LC tissues. Notably, heightened PFKL expression, driven by miR-128 downregulation, serves as a predictor for poor overall survival in patients. These findings strongly suggest that miR-128 may function as a tumor suppressor in LC by modulating cancer cell glycolysis [31].

In the context of miRNAs, miR-21 emerges as the key player in cancer progression. It exerts a profound influence mediated by cancer cells, undermining the effectiveness of conventional treatments [32]. The upregulation of miR-21 has been detected in various cancers, including LC [33]. Notably, miR-21 is overexpressed in both radiation-resistant cells and radiation-sensitive NSCLC cells after exposure to γ-irradiation. In such circumstances, miR-21 induces the upregulation of HIF1α, which contributes to the transcription of genes such as *LDHA* and *HKII*, thereby promoting glycolysis. The inhibition of glycolysis through the downregulation of miR-21 renders cells more radiosensitive [34]. Consistent with prior research findings, miR-21 was identified as significantly overexpressed in cisplatin-resistant NSCLC A549 cells. Its downregulation resulted in the inhibition of the PI3K/AKT/mTOR/HIF1 axis by reducing the levels of PKM2 and LDHA, which are two pivotal enzymes involved in glycolysis. Moreover, it was observed that the administration of cisplatin and a miR-21 sponge, either individually or in combination, led to distinct cellular responses. When administered alone, it induced autophagy, while in the case of combined treatment, it triggered autophagy, apoptosis, and necrosis concurrently [35]. Lastly, the involvement of miR-21 in regulating fructose-1,6-bisphosphatase 1 (FBP1), one of its potential targets, holds significant importance. FBP1 experiences downregulation in LC tissues and exhibits a negative correlation with miR-21. Additionally, the reversal of miR-21-induced effects by FBP1 overexpression suggests the emergence of a novel regulatory pathway in glucose metabolism, potentially unveiling a new molecular target [36].

## miRNA control of lipid metabolism

Lipids, including fatty acids, triglycerides, and cholesterol, play a significant role as a source of energy for metabolism in LC. Metabolites derived from fatty acids actively engage in the regulation of multiple genes, influencing energy production, membrane structure, signaling pathways, metastasis, and drug resistance [37–39].

Studies have demonstrated that miRNAs can influence metabolic enzymes, signaling pathways, or regulate transcription factors, either through direct or indirect mechanisms [11]. In line with this, Ni et al. show that elevating miR-21 levels facilitated cell migration and proliferation in human NSCLC cells. Furthermore, the cellular lipid content, encompassing phospholipids, neutral lipids, and triglycerides, significantly increased upon treatment with a miR-21 mimic compared with the control group. Key lipid metabolic enzymes, specifically fatty acid synthase (FASN), acetyl-coA carboxylase (ACC1), and fatty acid binding protein (FABP5), were prominently upregulated in human NSCLC cells.

Additionally, a relationship between miR-21 and CD36 has been suggested. CD36, also known as fatty acid translocase, plays a crucial role in lipid metabolism, facilitating the uptake of fatty acids by cancer cells from the extracellular matrix to support tumor growth. Suppressing CD36 expression mitigated the impact of miR-21 on cell growth, migration, and intracellular lipid accumulation. Furthermore, inhibiting miR-21 was found to suppress cell growth, migration, intracellular lipid levels, and CD36 protein expression in human NSCLC cells. The results also unveiled that peroxisome proliferator-activated receptor gamma co-activator 1-beta (*PPARGC1B*) was a direct target of miR-21, and reducing PPARGC1B levels reversed the decrease in CD36 expression caused by miR-21 inhibitor. These findings shed light on the mechanisms underlying the promotion of NSCLC by miR-21 and could offer novel therapeutic insights [40].

Among the most representative miRNAs in the regulation of lipid metabolism are the miR-15a-5p and the miR-421. Research has suggested that miR-15a-5p exerts its suppressive effect on lipid metabolism by inhibiting the activity of acyl-coenzyme A synthetase short-chain family member 2 (ACSS2) and reducing acetyl-CoA levels, thereby reducing the uptake of acetate for lipid synthesis. Furthermore, investigations unveiled that miR-15a-5p also hinders fatty acid synthesis through a reduction in histone acetylation at key sites (H4K8, H4K16, and H4K12) within the nucleus of tumor cells, particularly under hypoxic conditions. This discovery represents a significant contribution to the understanding of a novel mechanism for suppressing lipid metabolism and metastasis mediated by miR-15a-5p [41]. In both tumor tissues and NSCLC cell lines, miR-421 exhibited a significant increase, while *PTEN*, a tumor suppressor gene that regulates cell growth and survival, experienced a remarkable reduction. The downregulation of miR-421 was associated with decreased lipid accumulation, as well as diminished cell proliferation, migration, and invasion. Conversely, overexpression of miR-421 yielded opposite effects. Notably, miR-421 was identified as a direct regulator of PTEN, resulting in the negative modulation of its expression. Indeed, miR-421 activation led to the stimulation of the PI3K/AKT/mTOR pathway through PTEN regulation. This study unveils that miR-421 promotes lipid metabolism in NSCLC by targeting *PTEN* and activating the PI3K/AKT/mTOR pathway. These findings suggest that miR-421 may hold promise as a potential therapeutic target for NSCLC [42].

In conclusion, research has shown that specific miRNAs can modulate lipid metabolism in LC cells, exerting either promotional or inhibitory effects on lipid accumulation and metabolism by targeting key molecules and pathways. This knowledge provides a promising opportunity for therapeutic intervention.

## miRNA impact on amino acid metabolism

Amino acid metabolism is a crucial process in cancer cells as it contributes significantly in generating energy, synthesizing nucleosides, and ensuring the balance of redox reactions [43].

Although various studies have showcased the involvement of miRNAs in controlling amino acid metabolism, such as miR-23b* in kidney and breast cancer [44], and miR-499, miR-208b, and miR-23a in skeletal muscle, their roles in LC remain largely unexplored. Investigating the expression patterns and functional effects of miRNAs such as miR-23b*, miR-499, miR-208b, and miR-23a in LC cells could reveal novel insights into the molecular mechanisms driving LC progression and potentially identify new therapeutic targets for this deadly disease.

Noteworthy is the role of miR-126 in the context of LC. In SCLC cells, miR-126 targets *SLC7A5*, a key component of the amino acid transporter, associated with cancer aggressiveness. In these cells, reducing SLC7A5 expression, either through its suppression or miR-126 overexpression, inhibits cell proliferation by delaying progression through the G1 phase of the cell cycle. This suggests that miR-126 impacts the cell cycle partially through the regulation of SLC7A5. Consequently, reduced miR-126 levels contribute to increased SLC7A5 expression in SCLC cells, facilitating the efficient transport of essential amino acids required for rapid tumor cell growth [45]. Furthermore, miR-126 might also directly influence the cell cycle regulation in SCLC. The involvement of SLC7A5 in glutamine–leucine exchange leads to the activation of mTOR, a kinase that integrates nutrient and growth factor signals. This activation results in the phosphorylation of p70S6 kinase 1 and 4EBP1, ultimately stimulating the synthesis of growth-promoting proteins [46].

Given the established roles of miRNAs in regulating amino acid metabolism in various other cancers and tissues, it is plausible to hypothesize that specific miRNAs may also play significant roles in modulating amino acid metabolism in LC. Further research is needed to explore and validate the involvement of these miRNAs in LC and to assess their potential implications for diagnosis and treatment.

## miRNA-dependent redox regulation

Oxidative stress is due to increased intracellular levels of reactive oxygen species (ROS), which could damage lipids, proteins, and DNA. Maintaining ROS homeostasis is crucial for cell survival and signaling, both in physiological and pathological conditions. Cancer cells typically show heightened ROS levels and diminished antioxidant activity. The unbalance in ROS levels could alter the expression of oncogenes, tumor suppressors, and transcription factors, as well as noncoding RNAs such as miRNAs. In later stages of tumor progression, ROS can facilitate to cancer cell dissemination by activating NF-κB and MAPK pathways [47]. However, when ROS exceed a certain threshold, they enhance cell cycle arrest and apoptosis. It is known that alterations in the physiological levels of miRNAs can contribute to oxidative damage. ROS and miRNAs may reciprocally regulate each other to maintain ROS levels, thereby influencing cancer development [48].

In LC, decreased expression levels of miR-99a have been observed and are associated with metastasis, increased cell proliferation, and migration [35]. Under physiological conditions, miR-99a exerts its anti-metastatic function by targeting *NOX4*, a member of the NADPH oxidase (NOX) family [49], which promotes cell proliferation and migration by inducing ROS production. Therefore, miR-99a inhibits cancer progression by decreasing ROS levels, leading to the downregulation of MMP2 and MMP9, enzymes involved in extracellular matrix remodeling, often dysregulated in LC, which contribute to tumor invasion, metastasis, and angiogenesis [49].

miR-506 is highly expressed in the vast majority (83%) of patients with LC, and its expression is dependent on oxidative stress. It is well established that miR-506 negatively regulates NF-κB p65 expression, leading to increased ROS production. This elevated ROS level should activate p53, promoting cancer cell apoptosis [50].

However, it has been proven that ROS accumulation in cells with increased levels of miR-551b confers them apoptosis-resistance ability. Indeed, miR-551b inhibits the expression of catalase, resulting in ROS accumulation. This ROS accumulation activates the mucin-1 oncogene, thereby promoting increased survival and chemoresistance in LC cells [51].

**Fig. 1:**
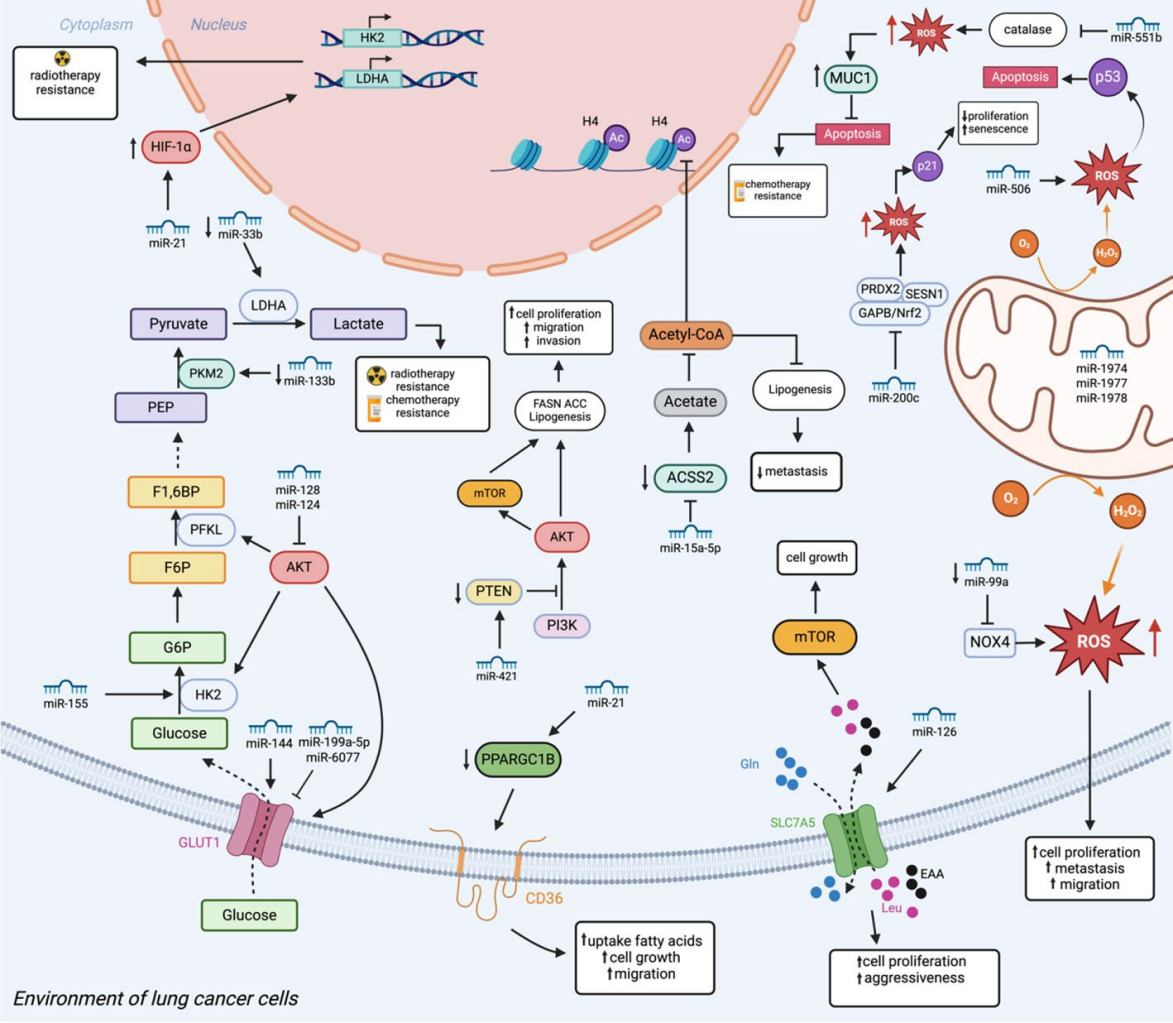
miRNA network regulating lung cancer metabolism. MiRNAs play a pivotal role in influencing the metabolic alterations observed in lung cancer cells by regulating signaling pathways and transcription factors. They have the capacity to modulate cell metabolism by targeting various components, such as glucose transporters (GLUTs) and key enzymes involved in glycolysis, including hexokinase 2 (HK2) and pyruvate kinase M2 (PKM2). Their regulatory effects on these glycolytic pathways are noteworthy. Additionally, activation of the PI3K/AKT/mTOR pathway can lead to enhanced lipogenesis. MiRNAs and mitochondrial miRNAs (mitomiRs) have been identified as crucial players in promoting mitochondrial metabolism and reactive oxygen species (ROS) production, acting both as oncogenes or tumor suppressors depending on the context. Furthermore, miRNAs have the ability to regulate amino acid metabolism. The miRNA-mediated metabolic rewiring strongly impacts cell malignancy and resistance to therapy. This Figure is generated with Biorender.com

Similarly, augmented expression of miR-200c in LC can regulate redox proteins, impinging on the level of intracellular ROS. miR-200c exerts its function by targeting the oxidative stress response genes, including *PRDX2*, *GAPB/Nrf2*, and *SESN1*, leading to enhanced ROS accumulation, and subsequently, increased p21 expression [52]. A summary of the miRNA-mediated metabolic rewiring in LC and a list of the main miRNAs involved are provided in Fig. 1 and Table 1, respectively.

**Table 1:** MicroRNAs involved in LC metabolic rewiring

miRNA	Target (direct and indirect)	Function	References
miR-133b	PKM2	Inhibition of glycolysis	[17]
miR-155	HK2	Induction of glycolysis	[21]
miR-33b	LDHA	Inhibition of glucose metabolism	[22]
miR-6077	GLUT1	Inhibition of glucose metabolism	[25]
miR-199a-5p	SLC2A1/GLUT1	Inhibition of glucose metabolism	[26]
miR-144	GLUT1	Inhibition of glucose metabolism	[27]
miR-124	GLUT1, HK2	Inhibition of glucose metabolism	[28]
miR-128	PFKL	Inhibition of glucose metabolism	[31]
miR-21	HIF1a, LDHA, HKII	Induction of glycolysis	[34, 35]
miR-21	FBP1	Induction of glycolysis	[36]
miR-21	CD36, PPARGC1B	Induction of fatty acid metabolism	[40]
miR-15a-5p	ACSS2	Inhibition of lipid metabolism	[41]
miR-421	PTEN	Induction of lipid metabolism	[42]
miR-126	SLC7A5	Inhibition of essential aa transport	[45]
miR-99a	NOX4	Regulates ROS levels by downregulation of MMP2 and MMP9	[49]
miR-506	NF-kB p65	Increased ROS production	[50]
miR-551b	Catalase	Increased ROS production	[51]
miR-200c	PRDX2, Nrf2, SESN1	Increased ROS accumulation	[52]
miR-22-3p	MTHFD2, MTHFR	Inhibition of folate metabolism	[70]
miR-9-5p	SHMT1 MTHFD1L, MTHFD2	Inhibition of folate metabolism	[70]
miR-218-5p	SHMT1	Inhibition of folate metabolism	[70]

*PKM2* piruvate kinase isoenzyme 2, *HK2* hexokinase, *LDHA* lactate dehydrogenase, *GLUT1/SLC2A1* glucose transporter 1, *PFKL* phosphofructokinase, *FBP1* fructose-1,6-bisphosphatase, *CD36* fatty acid translocase, *PPARGC1B* peroxisome proliferator-activated receptor gamma coactivator 1-beta, *ACSS2* acyl-coenzyme A synthetase short-chain, *PTEN* phosphatase and tensin homolog, *SLC7A5* solute carrier family 7 member 5, *NOX4* NADPH oxidase 4, *NF-kB* nuclear factor kappa-light-chain-enhancer of activated B cells, *PRDX2* peroxiredoxin-2, Nrf2 nuclear factor erythroid-2-related factor 2, *SESN1* sestrin 1, *MTHFD2* mitochondrial bifunctional methylenetetrahydrofolate dehydrogenase/cyclohydrolase, *MTHFR* methylene tetrahydrofolate reductase, *SHMT1* cytosolic form of serine hydroxymethyltransferase, *MTHFD1L* mitochondrial monofunctional C1-tetrahydrofolate synthase, *MTHFD2* mitochondrial bifunctional methylenetetrahydrofolate dehydrogenase/cyclohydrolase

## mitomiRNAs in regulating metabolism

Within the spectrum of miRNAs, an intriguing subgroup called mitomiRs emerges as a distinctive entity. mitomiRs are distinguished by their localization within the mitochondria. These specialized mitomiRs assume particular importance in the intricate orchestration of mitochondrial functions and metabolic processes [53].

The majority of mitomiRs are generated from the nuclear genome, although a subset originates from mRNA molecules derived from the mitochondrial genome [54]. Notably, the presence and association of mitomiRs with mitochondria exhibit species- and cell-type-specific variations. mitomiRs have been identified in mitochondria across diverse tissues and cell types, exhibiting unique thermodynamic properties compared with conventional miRNAs. Furthermore, mitochondria serve as a distinct and exclusive reservoir of mitomiRs, a phenomenon demonstrated by various experimental studies [53, 55]. MitomiRs hold the capacity to fine tune systemic energy balance, oxidative capacity, ROS, and mitochondrial lipid metabolism by modulating gene expression and protein regulation. As a result, they play a critical role in maintaining mitochondrial homeostasis and contributing to the overall cellular metabolic balance, making them central figures in cellular physiology and homeostasis. Notably, three identified miRNAs—miR-1974, miR-1977, and miR-1978—have been localized within the mitochondrial genome [56].

These miRNAs have not been extensively investigated within the context of mitochondria and LC, despite their known expression in LC [57–59]. Therefore, there is potential to speculate about their roles in this specific context. Nonetheless, numerous nuclear-transcribed miRNAs have demonstrated functionality within the mitochondrion. Regrettably, none of these have been specifically explored in the context of LC. It is plausible to consider that some of their functions, which are often not fully elucidated, might be executed within this cellular compartment. Given their distinct regulatory role within mitochondria, these mechanisms could potentially have relevance to LC cellular metabolism. Consequently, there is a compelling need to direct research efforts toward mitomiRs studies.

## Therapeutic exploitation of miRNA-mediated metabolic rewiring

**Fig. 2:**
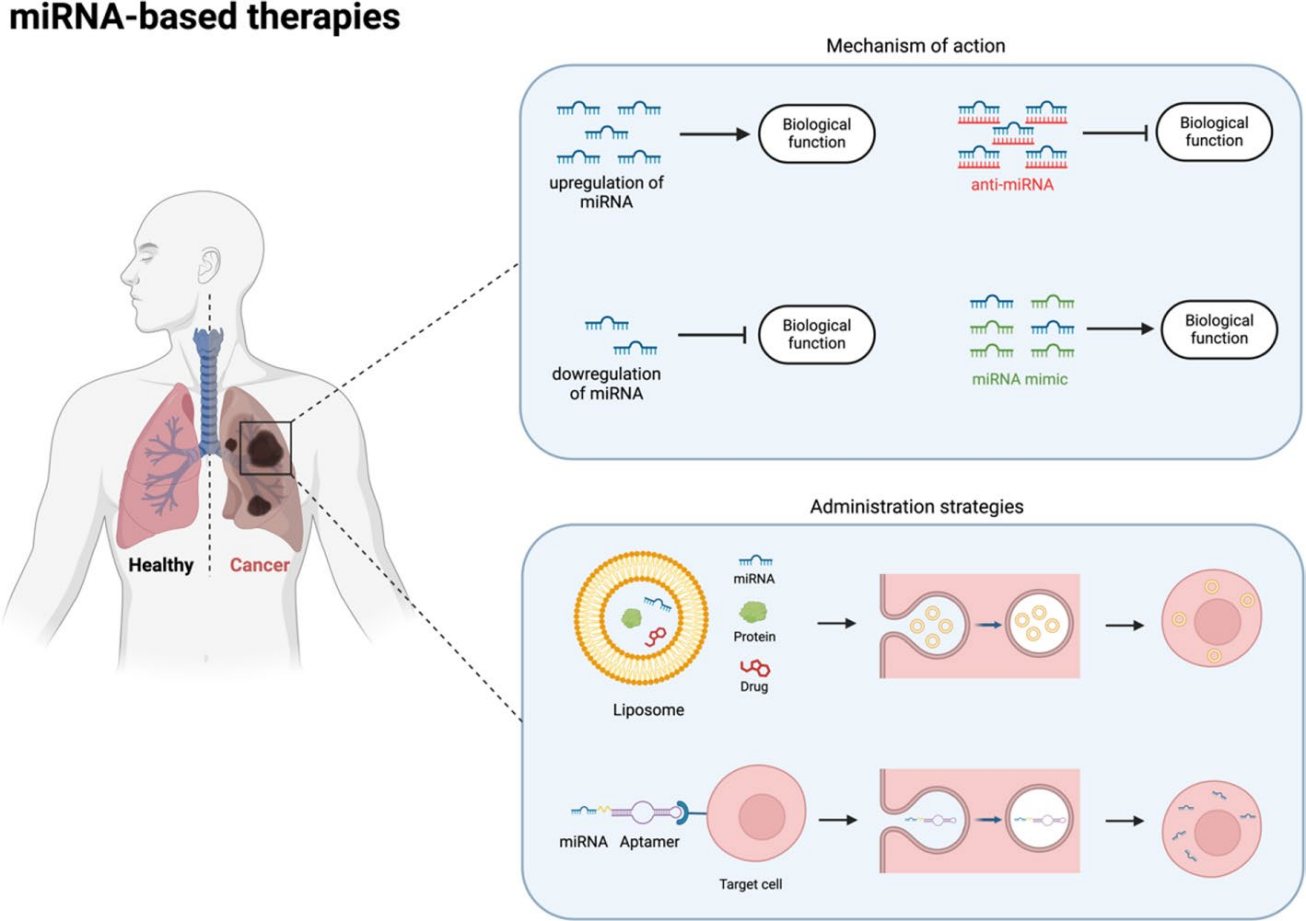
MiRNA-based therapeutics in lung cancer. Schematic representation of miRNA-based therapeutics in lung cancer. Anti-miRNA, miRNA mimic, and possible administration strategies including liposome and aptamer conjugated miRNA are illustrated. Anti-miRNAs inhibit the function of endogenous miRNAs, while miRNA mimics restore the function of tumor-suppressive miRNAs. Liposomes facilitate the delivery of miRNA-based therapeutics, and aptamer-conjugated miRNAs represent a novel approach for cell specific miRNA delivery with increased efficacy. This Figure is generated with Biorender.com

miRNAs exert a paramount role in the control of numerous functions inside the cells. They regulate signaling cascades involved in tumorigenesis [60], making them potential targets for therapy. miRNA-based therapy is designed to inhibit the expression of highly expressed miRNAs or to replenish insufficiently expressed ones. Inhibition could be reached using anti-miRs, which are oligonucleotides acting as antagonists to miRNAs, while replacement therapy can be obtained using miRNA mimics [61]. miRNAs play relevant functions inside tumor cells, but they also can be relevant for stroma cell education inside the TME. Released by both tumor and stroma cells, miRNAs can act as messengers, operating in a bidirectional way [9, 13]. Different miRNAs have been tested in vivo in LC models: miR-15/16 [62], miR-29b [63], miR-7 [64], miR-34a [65], let-7 [66], and miR-200c [52]. However, despite promising preclinical findings, only two phase I clinical trials have been conducted thus far. One of them involved the administration of MRX34, a liposomal mimic of miR-34a, to patients with advanced solid cancer (including NSCLC). Unfortunately, this phase 1 study was closed prematurely due to severe side effects, with a very low response rate of around 4% [67]. In contrast, a miR-16 mimic packaged in nanocells targeted with an anti-Epidermal Growth Factor Receptor (EGFR)-specific antibody (TargomiRs) has been tested in patients with advanced NSCLC. Encouragingly, this treatment was well tolerated, and after 8 weeks of administration, disease control was observed in five out of six treated patients [68, 69]. As mentioned, metabolic rewiring is a crucial process during tumor progression, with numerous miRNAs involved in the control of different aspects of the LC cell metabolism. As a result, metabolic-relevant miRNAs could be effectively harnessed to control and possibly revert the dangerous metabolic rewiring typical of aggressive tumor cells. In LC, miR-22-3p, miR-9-5p, and miR-218-5p were demonstrated to impact folate metabolism by targeting key enzymes involved in this pathway. By inhibiting folate metabolism, these miRNAs consequently influence the amino acid metabolome of LC cells. miR-22-3p in particular was shown to inhibit respiration and glycolysis and to reduce tumor growth in two xenograft mouse models derived from patients with NSCLC without causing any toxicity [70]. Preclinical and clinical miRNA-based therapies for LC are summarized in Table 2. However, miRNA-based therapies are still in their embryonic phase, facing numerous hurdles. Firstly, the multiplicity of the targets of miRNAs could give rise to off-target effects [71]. Secondly, restoration of miRNAs under-expressed during tumor progression via the administration of miRNA mimics may damage nontumor cells that uptake the administered miRNA [72]. Additionally, the use of engineered viral vectors for delivery may be associated with the risk of dangerous integration of viral DNA in the host genome [73]. Different attempts have been made to solve these issues and make miRNA delivery more specific. Charged liposomes and nanoparticles have been used but with contradictory results. In some cases, toxic effects were observed, while liposomes, for example, may be unstable and evade immune response [74]. Precise identification of target cells is crucial for the efficient and accurate delivery of miRNAs. Aptamers could be used for this purpose. They are a promising class of anticancer treatments whose main characteristic is the ability to recognize targets thanks to their three-dimensional structure [75]. Moreover, they can be used as highly efficient carriers for the transport of proteins, drugs, and nucleic acids, such as miRNAs. They can aid in obtaining a targeted delivery, thereby mitigating toxicity and reducing side effects due to the conjugated substance. Through association with aptamers, miRNAs can be selectively targeted to tumor cells [76, 77]. However, some challenges have emerged in the use of aptamers for systemic treatments. Among these challenges, their limited stability and high renal clearance are among the major issues, and huge efforts are put in place to improve delivery, such as through the use of nanoparticles or by adding chemical modifications such as poly(ethylene glycol) (PEG) into the aptamers structure. Besides the limitations described above, miRNA-based therapies are promising tools to tune LC metabolism to improve patients’ response to drugs (Fig. 2).

**Table 2:** Pre-clinical and clinical miRs in Lung Cancer

miRNA	Preclinical studies	Clinical trial phase	References
miR-15	Yes	–	[41]
miR-16	Yes	I	[68, 69]
miR-29b	Yes	–	[63]
miR-7	Yes	–	[64]
miR-34	Yes	I*	[65, 67]
let-7	Yes	–	[66]
miR-200	Yes	–	[52]
miR-22-3p	Yes	–	[70]
miR-9-5p	Yes	–	[70]
miR-218-5p	Yes	–	[70]

^*^Closed due to side effects

## Challenges and future directions

Numerous challenges persist in comprehending the role of miRNAs in LC. For instance, understanding how miRNAs participate in various subtypes of LC and their connection to subtype-specific metabolic changes poses a significant challenge. Additionally, unraveling the exact functions of individual miRNAs in regulating metabolic pathways within LC cells remains intricate and somewhat elusive. The integration of miRNA expression profiles with other omics data, including genomics, transcriptomics, and metabolomics, holds immense potential for achieving a more thorough understanding of the intricate regulatory networks governing metabolism in LC. An additional challenge lies in identifying dependable miRNA-based biomarkers for early diagnosis, prognosis, and predicting the response to primary targeted therapies with a metabolic focus.

Moreover, the endeavor to develop impactful therapeutic strategies by modulating miRNAs to target metabolic vulnerabilities in LC presents challenges related to delivery systems, potential off-target effects, and the risk of resistance. Finally, mitomiRs, a subclass of microRNA miRNA molecules, play a pivotal role in regulating the expression of mitochondrial proteins and overseeing the mitochondrial functional activity. Consequently, there is a keen interest in exploring the involvement of mitomiRs in the genesis of LC.

## Conclusions

In recent years, miRNA-related studies in the context of LC metabolism have yielded valuable insights with profound implications for therapeutic advancements. These investigations have shed light on the intricate relationship between miRNAs and metabolic pathways, offering promising avenues for innovative treatment strategies.

Numerous studies have revealed dysregulated miRNA expression patterns in LC cells, emphasizing their pivotal roles in metabolic reprogramming. miRNAs such as miR-21 and miR-144 have consistently been implicated in regulating key metabolic processes, including glycolysis, oxidative phosphorylation, and lipid metabolism.

Dysregulated miRNAs in LC have been associated with aggressive tumor phenotypes and poorer patient outcomes. These miRNAs often target critical metabolic enzymes, transporters, or regulators, leading to altered energy metabolism and increased proliferative capacity of cancer cells.

In addition to their cell-autonomous role, miRNAs can exert non-cell-autonomous effects. Once produced, they can affect other cells, interfering with components of the microenvironment. For example, the miR-155 can modulate the metabolic status of immune cells, affecting their functions within the tumor immune microenvironment. This interaction highlights the potential of miRNA-based therapies in immunomodulation. Several examples illustrate the potential to modulate miRNA expression in tumor cells or the TME, either directly in vivo through a combination of miRNA targeting and delivery carriers or by modifying immune cells in vitro before infusion.

Strategies aimed to restore or inhibit specific miRNAs have shown promise in preclinical models. miRNA-based therapeutics, including miRNA mimics and inhibitors, are being explored for their potential to rewire metabolic pathways in cancer cells. Combinatorial approaches that integrate miRNA-targeted therapies with conventional treatments, such as chemotherapy or immunotherapy, have demonstrated synergistic effects. miRNAs can sensitize cancer cells to existing therapies by modulating their metabolic vulnerabilities. Furthermore, miRNA profiling and identification of patient-specific miRNA signatures have the potential to guide personalized treatment strategies in LC. Tailoring miRNA-based interventions to an individual’s unique miRNA expression profile may enhance treatment efficacy.

Despite the promise of miRNA-based therapies, challenges remain, including efficient delivery methods and off-target effects. Ongoing research aims to address these issues and refine the therapeutic potential of miRNAs in LC metabolism.

Our review emphasizes the diverse roles played by various miRNAs in the pathogenesis and progression of LC, highlighting their significance as key regulators of metabolic processes. However, while there is ample research on the molecular activity of miRNAs, studies on their translational applicability remain limited. Regrettably, the exploration of miRNA and noncoding elements for precision medicine appears to have reached a standstill. The waning interest in miRNAs and noncoding elements may stem from evolving research trends or challenges in translating discoveries into clinical applications. Regardless of the specific cause, delving into the dark matter of DNA, which constitutes a major portion of the genome, presents a profound opportunity in molecular medicine. A comprehensive exploration not only at the molecular level, but also from a therapeutic and diagnostic perspective, could unveil valuable insights and potential advancements.

## Data Availability

Not applicable.

## Abbreviations

PKM2: Pyruvate kinase isoenzyme M2
NSCLC: Non-small cell lung cancer
SCLC: Small cell lung cancer
GLUT1: Glucose transporter 1
LDHA: Lactate dehydrogenase A
AC: Adenocarcinoma
FASN: Fatty acid synthase
ACC1: Acetyl-CoA carboxylase 1
FABP5: Fatty acid binding protein 5
PTEN: Phosphatase and tensin homolog
FBP1: Fructose-1,6-bisphosphatase 1
ROS: Reactive oxygen species
NOX4: NADPH oxidase 4
MMP2: Matrix metalloproteinase 2
MMP9: Matrix metalloproteinase 9
PPARGC1B: Peroxisome proliferator-activated receptor gamma coactivator 1-beta
miRNA: MicroRNA
TME: Tumor microenvironment
